# *Bacillus cereus* AR156 Extracellular Polysaccharides Served as a Novel Micro-associated Molecular Pattern to Induced Systemic Immunity to *Pst* DC3000 in *Arabidopsis*

**DOI:** 10.3389/fmicb.2016.00664

**Published:** 2016-05-09

**Authors:** Chun-Hao Jiang, Zhi-Hang Fan, Ping Xie, Jian-Hua Guo

**Affiliations:** Key Laboratory of Monitoring and Management of Crop Diseases and Pest Insects, Department of Plant Pathology, College of Plant Protection, Ministry of Agriculture, Engineering Center of Bioresource Pesticide in Jiangsu Province, Nanjing Agricultural UniversityNanjing, China

**Keywords:** induced systemic resistance (ISR), non-host resistance, micro-associated molecular patterns (MAMPs), extracellular polysaccharides, *Bacillus cereus* (AR156), biological control

## Abstract

Non-host resistance (NHR) is a broad-spectrum plant defense. Upon colonizing on the surface on the root or leaves of non-host species, pathogens initial encounter preform and induce defense response in plant, such as induced hypersensitive response, PAMPs triggered immunity (PTI), and effector triggered immunity (ETI). The ability of plants to develop an induced systemic response (ISR) in reaction to the colonization by non-pathogenic rhizobacterium depends on interactions between host plants and the colonizing rhizobacterium, and the ISR also can be defined as a NHR. However, how the colonization signal is and how systemic resistance to pathogens is developed is still unclear. In this study, we demonstrated that the extracellular polysaccharides (EPSs) of *Bacillus cereus* AR156 could act as novel microbe-associated molecular patterns (MAMPs) and function in the early perception status of the ISR of *B. cereus* AR156. The results revealed that *B. cereus* AR156 EPS could induce systemic resistance to *Pst* DC3000 in *Arabidopsis*. Cellular defense response markers such as hydrogen peroxide accumulation, callose deposition, and defense-associated enzyme were induced upon challenge inoculation in the leaves primed by EPS. Moreover, the defense-related genes *PR1, PR2*, and *PR5* and mitogen-activated kinases (MAPK) cascade marker gene *MPK6* were concurrently expressed in the leaves of EPS-treated plants and induced higher resistance to *Pst* DC3000 in Col-0 than that in the *jar1* or *etr1* mutants. The protection was absent in the *NahG* transgenic plants and *npr1* mutant, suggesting an activation of the salicylic acid (SA)- and the MAPK-dependent signaling pathways with NPR1-dependent by *B. cereus* AR156 EPS. In conclusion, *B. cereus* AR156 EPS play an important role in MAMP perception during the process of rhizobacteria-triggered NHR. This study is the first to illustrate how AR156 induces systemic resistance to *Pst* DC3000 in *Arabidopsis*. It also provides the first explanation of how plants perceive colonization of non-pathogenic bacteria and how rhizobacteria trigger ISR to plant pathogens.

## Introduction

Plant growth is influenced by a variety of biotic and abiotic factors. To survive from an antagonistic and complex environment, the plant has evolved a series of inducible defense mechanisms, which can assist them to activate appropriate defense reactions upon pathogen invasion (Niu et al., 2011; Jiang et al., 2015). Well-researched examples of plant-induced resistance include SAR and rhizobacteria-ISR, which are phenotypically similar to each other and both of them can be defined as a non-host resistance (NHR) (Conrath et al., 2002; Jiang et al., 2015). NHR is one kind of resistance exhibited by an entire plant species to all genetic variants of a non-adapted pathogen species in nature. Phyto-hormones such as SA, JA, and ethylene play important but quite different roles in the signaling network, which function on regulating the development of ISR and SAR (Glazebrook, 2001; Niu et al., 2011). As previously reported, the SAR is induced by SA, and its onset involves both systemic and local increases in endogenously synthesis of phyto-hormones SA, leading to activation of pathogenesis-related (PR) proteins encoding genes, such as *PR1*, *PR2*, and *PR5* (Ward et al., 1991; Van Loon and Van Strine, 1999; Niu et al., 2011; Jiang et al., 2015). In contrast, ISR requires the JA and ET signaling pathways (Van Loon et al., 1998; Jiang et al., 2015) and is combined with the high expression of the plant defensin 1.2 (*PDF1*.*2*) (Van Oosten et al., 2008; Jiang et al., 2015).

Induced systemic response has been found and demonstrated in series of plant species [e.g., cucumber (*Cucumis sativus*), bean (*Phaseolus vulgaris*), and tomato (*Solanum lycopersicum*) (Van Loon et al., 1998; Van der Ent et al., 2008; Jiang et al., 2015)]. The rhizobacterium *Pseudomonas fluorescens* WCS417r (WCS417r hereafter) has been demonstrated, which could trigger ISR in series of plant species (Pieterse et al., 2002). Nevertheless, it has also been documented that *Bacillus cereus* AR156 induces systemic resistance in *Arabidopsis* (Niu et al., 2011). The ability of plants to trigger ISR in response to the root colonization by non-pathogenic rhizobacterium relies on the interactions between host plants and the rhizobacterium (Van Loon et al., 1998; Pieterse et al., 2002), which rises following questions: How do plants perceive the colonization of non-pathogenic bacteria? How do bacteria colonizing plant roots trigger ISR to plant pathogens in systemic tissue?

Plants are equipped with a multiple of immune receptors, which can sense the invasion of numerous pathogenic microbes (Boller and Felix, 2009; Dodds and Rathjen, 2010; Monaghan and Zipfel, 2012; Spoel and Dong, 2012; Li et al., 2014). At the front line, plants have two main modes functions on pathogen recognition. The first mode was pattern recognition receptors (PRRs), which localized on the plasma membrane and function on the recognition of pathogen/microbe-associated molecular patterns (PAMPs/MAMPs). Most of these conserved signatures are belongs to the essential microbial structures, such as flagellin of bacteria, cell walls and translation factors (Boller and Felix, 2009; Mari-Anne et al., 2013; Newman et al., 2013; Sreekanta et al., 2015; Trdá et al., 2015). The endogenous signals may cause similar responses to pathogen, which was called damage-associated molecular patterns (DAMPs). These signals include oligogalacturonides (OGs) and cutin monomers, which could act as a critical component of signaling in signaling tranduction (Kauss et al., 1999; Huffaker and Ryan, 2007; Yamaguchi et al., 2010; Rasul et al., 2012; Bartels et al., 2013; Sreekanta et al., 2015).

Host resistance responses induced by MAMPs are collectively referred to as pattern-triggered immunity (PTI) (Jones and Takemoto, 2004; Zhang and Zhou, 2010). In pattern-triggered immunity, the defense responses are motivated following by the recognition of pathogen PAMPs, such as flg22, which is a conserved 22 amino acid N-terminal sequence of the bacterial flagellin protein. In *Arabidopsis*, flg22 has been well studied and perceived by PRRs such as FLAGELLIN-SENSITIVE 2 (FLS2). Recent studies have shown that PTI also plays an important role in NHR. For example, the flagellin of biocontrol strain, which can act as MAMPs and recognized by PRRs also can trigger ISR. Meziane and asscociate reporte that the isolated flagellin of *P. putida* WCS358 was shown to trigger ISR against *Pst* DC3000 in *Arabidopsis*, as well as an Lipopolysaccharides (LPS) from *P. fluorescens* WCS417r and *P. putida* WCS358 (Meziane et al., 2005). Beside the flagellin, the bacterial Lipopolysaccharides also can act as a prototypical PAMP which can induce series of plant defense-related responses, such as the nitric oxide (NO) generation, oxidative burst, the cell-wall alteration, callose deposition, and the expression of *PR* gene (Sun and Li, 2013).

Zhang et al. (2007) and associates reported that the reactive oxygen species (ROS) production in the process of PTI was mediated primarily by the NADPH oxidase RBOHD, which was necessary for callose deposition (Torres et al., 2002; Sreekanta et al., 2015). The following responses included production of SA, JA, and ET, the expression level of defense-related gene changes (van Loon et al., 2006; Browse, 2009; Vlot et al., 2009; Sreekanta et al., 2015). Many studies have reported that phyto-hormones SA, JA, and ET play important roles in the signaling network on regulating the development of ISR. As we all known, some beneficial rhizobacteria can trigger ISR by priming the plant for potentiated activation of several cellular defense responses. The potentiated responses include oxidative burst, callose deposition (Iriti et al., 2003), defense-related enzymes accumulation (Benhamou and Belanger, 1998), and some secondary metabolites production (Yedidia et al., 2003). Therefore, here we raised a question: Is there a crucial part of microbial structures that could act as a type of MAMP that could be perceived by plants and trigger ISR to the pathogen?

Microorganisms have evolved in various responses to biotic and abiotic stress and interact with their environments (Pintor et al., 2012; Singh et al., 2012; Huang and Liu, 2013). An efficient response mechanism is the EPS hereafter secretion (Wei et al., 2011; Wang et al., 2014). A series of studies have reported that EPS act as highly potent and efficient signatures that can perceive environment signals and interact with plants. We propose that the EPS of rhizobacteria can act as MAMPs and facilitate the induction of systemic resistance in some rhizobacteria to pathogens in plants.

We previously isolated the rhizobacterium *B. cereus* strain AR156. In our previous study, it was found that colonization of *Arabidopsis* roots by AR156 could enhance resistance against a broad-spectrum disease. We further demonstrated that AR156 could elicit ISR through simultaneously activating of the SA and JA/ET two signaling pathways (Niu et al., 2011). To further investigate how plants perceive the colonization of non-pathogenic bacteria and how bacteria trigger ISR to plant pathogens, we carried out the current study by using a well-established system employing ISR-inducing *B. cereus* strain AR156 and a plant-pathogen interacting system (*Arabidopsis* and *Pst* DC3000), a common practice in our lab over the past few years (Ref).

In this study, we demonstrated that *B. cereus* AR156 EPS could act as novel MAMPs during rhizobacterium-ISR that functioned to distinguish between the rhizobacterium and plant. Results showed that the *B. cereus* AR156 EPS could be perceived by *Arabidopsis*, which activated the downstream immune response, including the activation of various cellular defense responses and changes in gene expression encoding PR proteins [*PR1, PR2, PR5*] through the MAPK- and SA-signaling pathways. The *B. cereus* AR156 EPS-triggered ISR was dependent on an ankyrin repeat protein, NPR1 (non-expresser of PR genes 1)/NIM1 (non-inducible immunity). This study is the first to illustrate and provide a clear explanation regarding how the plant perceives the colonization of non-pathogenic bacteria and triggers ISR to plant pathogens when the pathogens are localized on the surface of the plant root.

## Materials and Methods

### Plants, Bacterial Strains, and Growth Conditions

The following *Arabidopsis* lines were used: Col-0 (wild-type *Arabidopsis thaliana* ecotype); *npr1* (Bowling et al., 1994); signaling mutants *jar1* (Staswick et al., 1992); and *etr1* (Bleecker et al., 1988) and a transgenic line *NahG* (overe-expressing the bacterial *NahG* gene in Col-0) (Delaney et al., 1994); All Seeds of *Arabidopsis* lines were sown in an soil combined with sterilized vermiculite and potting soil, and the 2-week-old seedlings were transferred into 200 ml pots, which filled with a mixture of sterilized vermiculite and potting soil, each pot placed one seedling. All the plants were cultivated in a growth chamber with a suitable condition, which was 10 h day (200 μE m^-2^ s^-1^ at 22°C) and a 14 h night (20°C) cycle at 70% relative humidity and the whole growth process was supplied with modified half-strength Hoagland nutrient solution weekly (Hoagland and Arnon, 1938).

The studied PGPR strain *B. cereus* AR156 was cultivated on LB (Luria-Bertani) (yeast extract 5 g, peptone 10 g, NaCl 10 g in 1 L of water) agar plates at 28°C for 24 h. The *B. cereus* AR156 cells were pelleted by centrifugation and re-suspended in sterile 10 mM MgCl_2_ solution and then adjusted density to 5 × 10^7^ CFU ml^-1^ for use.

The challenging pathogen *Pst* DC3000 was cultivated in the liquid King’s B medium (peptone 20 g, K_2_HPO_4_1.5 g, MgSO_4_.7H_2_O 1.5 g, Glycerol 10 ml, in 1 L of water), which containing 50 mg/L of rifampicin at 28°C overnight. The *Pst* DC3000 cells were collected by centrifugation and re-suspended in 10 mM MgCl_2_ solution, in which containing 0.01% (v/v) of the surfactant Silwet L-77 (bought from Sigma, St Louis) and adjusted density to 5 × 10^7^ CFU ml^-1^ for use (modified from Niu et al., 2011).

### Extraction and Purification of Extracellular Polysaccharides from *B. cereus* AR156

The extraction and purification of EPS from *B. cereus* AR156 cells was carried out by reference to the method of Hung et al. (2005). The *B. cereus* AR156 cells were inoculated into 1 L of LB liquid medium within 48 h after it had been autoclaved at 121°C for 20 min. After allowing the culture to grow for 48 h, an aliquot from the bacterial culture was taken and centrifuged at 5000 g for 30 min. After centrifugation, the culture contained two layers: a pellet and a supernatant. EPS were then extracted from the supernatant. Five volumes of isopropanol were added to precipitate EPS overnight at 4°C. The next day, the pellets were spun down, re-suspended in 10 ml of freshly made digestion mix (0.1 M MgCl_2_, 0.1 mg/ml RNase and 0.1 mg/ml DNase in ddH_2_O), and incubated for 1 h at 37°C. The treated samples were extracted with equal volumes of phenol:chloroform twice. The samples were then collected and dialyzed in a large volume of ddH_2_O (for example 5L) for 24–48 h at room temperature. The dialyzed samples were collected and dried using a lyophilizer (LyoQuest^TM^ -85). A 50 mg/ml EPS aqueous solution was prepared for use in the following experiments (Hung et al., 2005; Dogan et al., 2015).

### Hypersensitive Response Analysis

In this study, we employed two systems to perform a hypersensitive analysis: *Arabidopsis* and tobacco. In *Arabidopsis*, the left half of the leaves, which was from 6-weeks-old *Arabidopsis* plants were infiltrated with *B. cereus* AR156 EPS (50 mg/ml); The *Pst* DC3000 strains which containing vector only or overexpressing of *avrRpt2* (1 × 10^7^ CFU ml^-1^), the supernatant of *B. cereus* AR156, and the cell suspension of *B. cereus* AR156 (5 × 10^7^ CFU ml^-1^). MgCl_2_ (10 mM) and LB liquid medium were employed as negative controls. Leaves were stained with Trypan Blue 6, 14 or 20 hpi as previously described (Koch and Slusarenko, 1990; Katagiri et al., 2002; Takahashi et al., 2003). The same treatments and methods were used in tobacco. All experiments were performed three times.

### Antagonistic Analysis of Extracellular Polysaccharides of *B. cereus* AR156 to *Pst* DC3000 *In Vitro*

In this study, the antagonistic activities of *B. cereus* AR156 EPS and the *B. cereus* AR156 component were assayed. A 10 mL suspension of 1.0 × 10^7^ CFU/mL *P*st DC3000 strains containing vector only or clones expressing *avrRpt2* were added to 1 L of King’s B medium (make sure the temperature lower than 50°C), and mixed completely, then poured into plates. A 50 mg/ml EPS aqueous solution was prepared and added into an Oxford cup; the solution was then placed onto a plate containing previously prepared *P*st DC3000 and incubated at 28°C for 2 days. This procedure was repeated three times for each treatment. The supernatant of *B. cereus* AR156, LB medium, and sterile water were used as controls. For the *B. cereus* AR156 itself, the strain was placed onto the plates and incubated at 28°C for 2 days. Four repeats were set on one plate. Finally, the diameter of the clear semicircular hyaline zone, which was surrounding the bacterial plaque, was measured after incubation, and the size of the clear semicircular hyaline zone was used to evaluate the antagonistic activities.

### Induction Treatments

Six-weeks-old plants were subjected to bacteria induction treatments in 4 weeks later. For the *B. cereus* AR156 EPS treatments, 50 mg/ml EPS was sprayed onto the leaf surface of the *Arabidopsis* ecotype Col-0 plant. For the control treatment, sterilized water was sprayed onto the leaf surface of the *Arabidopsis* ecotype Col-0 plant.

Five days after the induction treatment, half of the seedlings in each treatment were challenge-inoculated through spraying the cell suspension of the virulent pathogen *Pst* DC3000 (5 × 10^7^ CFU ml^-1^) on the leaves, until all the leaves were covered with fine droplets. The other halves of the remaining plants were sprayed with 10 mM MgCl_2_ as the negative control. All the plants were incubated in a dew chamber with 100% relative humidity for 3 days and then transferred to a normal growth chamber after challenge inoculation.

### Density Detection of *Pst* DC3000 in *Arabidopsis* Leaves

To detect the density of *Pst* DC3000 in *Arabidopsis* leaves, samples were collected at three time points: 0, 3, and 4 days post inoculation (dpi). *Arabidopsis* leaves (0.1 g) were surface sterilized by 70% ethanol, then washed in sterilized water for three times, and then homogenized by using a sterilized mortar and pestle with 0.9 ml 10 mM MgCl_2_. Subsequently, suitable dilutions were plated onto King’s B agar containing with 100 mg l^-1^ cycloheximide and 50 mg l^-1^ rifampicin (Pieterse et al., 1996), then incubated at 28°C. After 48 h, the colonies shown on the plate were counted, and then the density of *Pst* DC3000 in the *Arabidopsis* leaves was detected, expressed as CFU g^-^1 fresh leaf (FW). This experiment was repeated three times. Means were compared by using a LSD test (short of least significant difference) (*P* = 0.05), then the LSD results and standard errors and were calculated (Niu et al., 2011; Jiang et al., 2015).

### Detection of Hydrogen Peroxide Accumulation and Activities of SOD and POD

The content of hydrogen peroxide and the activities of SOD and POD in plants were determined according to the method of Jiang et al. (2015) using a ‘Hydrogen Peroxide assay kit’, ‘SOD detection kit’, and ‘POD detection kit’, respectively, (Nanjing Jiancheng Biological Engineering Institute, Nanjing, China) according to the manufacturer’s instructions (Jiang et al., 2015).

To experimental methods used for determining the accumulation of hydrogen peroxide and callose deposition in different treatments were referred from the methods described by Reuber et al. (1998) and Niu et al. (2011). There was no change in our study.

### Plant RNA Extraction and RT-PCR Analysis

*Arabidopsis* leaves for RT-PCR analysis were soaked in liquid nitrogen. The total RNA of each sample was extracted by the TRIZOL reagent (Invitrogen, Cat. No. 15596-026), referring to the manufacturer’s recommendations. RT-PCR was developed with 1 mg total RNA, treated with DNase I (gDNA Wiper from Vazyme^TM^, Cat. No. R133-01). Reverse transcription was conducted using HiScript^TM^ Q Select RT SuperMix (Vazyme^TM^, Cat. No. R133-01). Quantitative RT-PCR was conducted on an ABI 7500 system (ABI) using the SYBR premix Ex-Taq mixture (Takara). PCR was performed under the following conditions: 94°C for 5 min, followed by 45 cycles of 94°C for 10 s, 55°C for 20 s, and 72°C for 30 s, and end with 72°C for 5 min. *At-BETA-TUB 4* (locus: *At4g44340*) was employed as the internal standard. All the PCR primers used in our study are listed in **Supplementary Table S1**.

### Protein Extraction and Western Blotting Analysis

To detect the MAPK6 protein expression level during the EPS-ISR process, samples were collected at 3 and 5 days post-EPS treatment (dpt) and at 6 and 12 h post *Pst* DC3000 inoculation (hpi). *Arabidopsis* leaves were soaked in liquid nitrogen, and the total protein in the leaves was extracted by a homemade protein extraction buffer (Zhang et al., 2011). The protein concentration was detected by using Bradford reagent (Bio-Rad). 10 μg of total protein was size-fractionated by SDS–PAGE gel for Western blotting analysis, then all the proteins were transferred to a Hybond-P PVDF membrane (bought from GE Healthcare) in transfer buffer in which containing 10% methanol. The membrane was probed with a specific MAPK6 antibody (purchased from Sigma–Aldrich, Cat. No. HPA030262) and a secondary horseradish-peroxide-conjugated antibody (anti-rabbit-HRP) purchased from Sigma–Aldrich (Cat. No. R2004). The final antibody-protein complexes were determined by using ECL-plus (GE Healthcare) on a Bio-Rad Versa doc 5000, and the image was analyzed using Quantity One software. β-Tubulin was used as an internal standard. The β-tubulin antibody was purchased from Sigma–Aldrich (Cat. No. T2200).

### Analysis of the Characteristics and Composition of Extracellular Polysaccharide

Finally, the IR and UV spectra, the molecular weight and the monosaccharide composition of the sample were obtained to analyze the characteristics and composition of *B. cereus* AR156 EPS. The IR spectrum analysis was carried out by using an FTIR, Jasco 6100, Model Japan (resolution: 4 cm^-1^). The sample was ground with spectroscopic grade potassium bromide powder and then pressed into a 1 mm pellet for FTIR measurement over the frequency range of 4000–400 cm^-1^ (Mid infrared region). The UV spectrum of the EPS was recorded by using a T80+UV/VIS Spectrometer, PG Instrument Ltd. (range: 190–1000 nm). The molecular weight and monosaccharide composition assays were performed as described by Haroun and associates (Haroun et al., 2013).

## Statistical Analysis

All bioassays were conducted three times with 24 seedlings per treatment. Analysis of variance (ANOVA) was carried out using SPSS software version 19.0 (IBM). A mean comparison was conducted by using LSD test (*P* < 0.05). Standard errors and standard deviations (SDs) were calculated.

## Results

### The Extracellular Polysaccharides of *B. cereus* AR156 Elicited Strong HR in Plants Leaves

**FIGURE 1:**
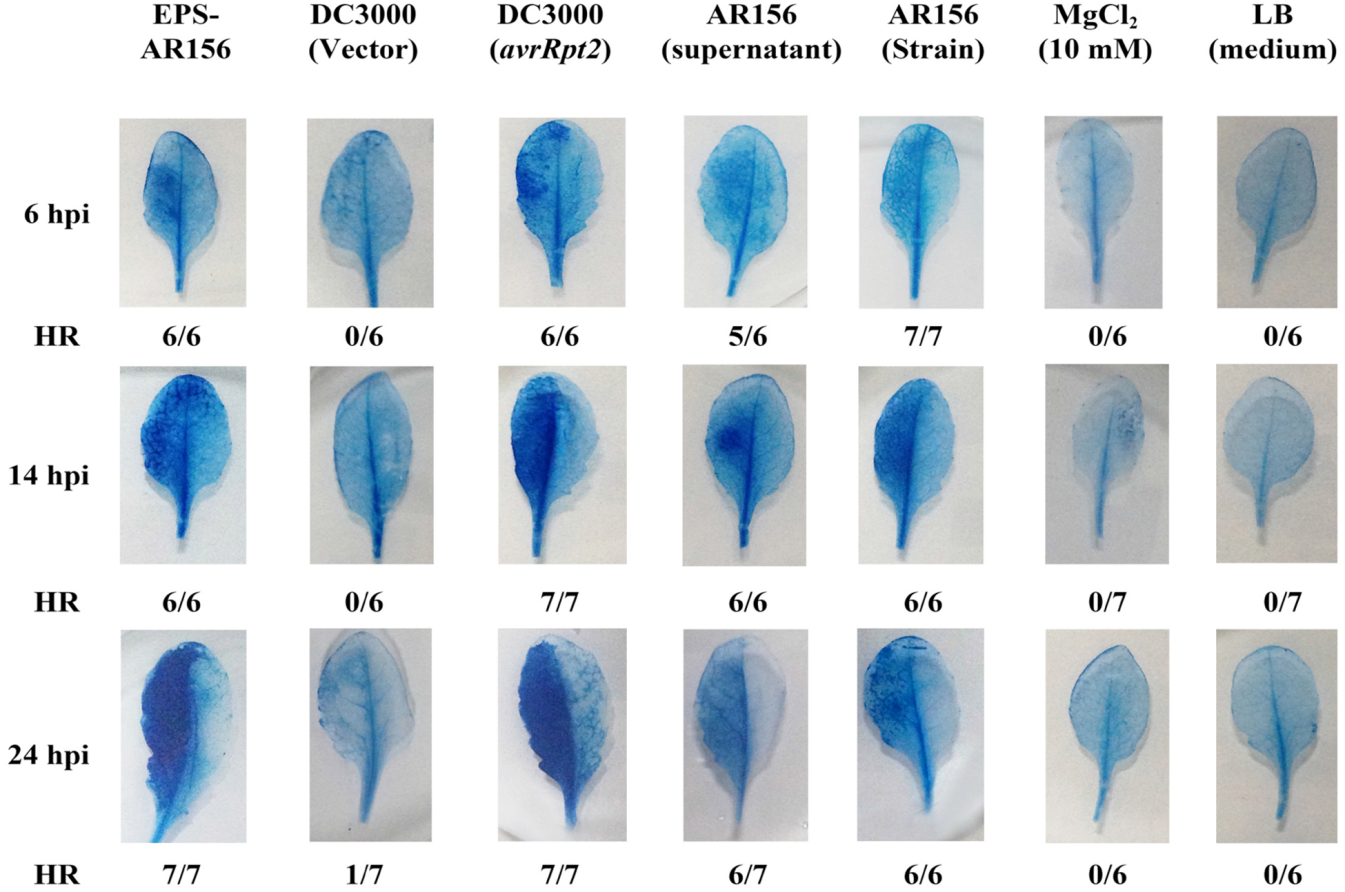
**The EPSs of *Bacillus cereus* AR156 elicited strong HR in non-host *Arabidopsis* ecotype Col-0 plants leaves.** The HR test was conducted using Trypan Blue staining. The left half of the leaves from 6-week-old *Arabidopsis* ecotype Col-0 plants were infiltrated with *B. cereus* AR156 EPS (50 mg/ml); *Pst* DC3000 strains (1 × 10^7^ CFU ml^-1^) containing vector only; or clones expressing *avrRpt2*, the supernatant of *B. cereus* AR156, and the cell suspension of *B. cereus* AR156 (5 × 10^7^ CFU ml^-1^). MgCl_2_ (10 mM) and LB liquid medium were employed as negative controls. Leaves were stained with Trypan Blue at 6, 14, or 20 hpi. Fractions indicate the number of leaves exhibiting HR and the total number of leaves tested. hpi, hours post-inoculation. All experiments were performed three times, and similar results were obtained.

In our previous study, *B. cereus* AR156 was found to not only promote the growth of *Arabidopsis* but also trigger ISR (Niu et al., 2011). To demonstrate how *B. cereus* AR156 was perceived by plants and how ISR was triggered in plants, a series of components of *B. cereus* AR156 were examined and tested in this study. As shown in **Figure 1**, the *Arabidopsis* leaves of the *B. cereus* AR156 EPS treatment started to show HR symptoms 6 h post infiltration (hpi), and the symptoms became more significant at 12 h (**Figure 1**). The same symptoms were observed in *B. cereus* AR156 and its supernatant treatment. In this study, we also used *Pst* DC3000 strains containing vector only or clones expressing *avrRpt2* as a control. The results described in a previous study are shown in **Figure 1**(Takahashi et al., 2003). To confirm these results, we employed a tobacco system to repeat the experiments. As shown in Figure S1, the *B. cereus* AR156 EPS could significantly elicit HR on tobacco leaves (**Supplementary Figure S1**). The *B. cereus* AR156 could trigger HR on non-host plants in a manner resembling that observed for the pathogenic *P. syringae* strain *Pst* DC3000. *B. cereus* AR156 is avirulent to plants such as *Arabidopsis* (data not shown). Therefore, we concluded that *B. cereus* AR156 might act as a beneficial elicitor that could be perceived by plants and could trigger immunity similarly to pathogens.

### The Extracellular Polysaccharides of *B. cereus* AR156 Triggered ISR to *Pst* DC3000 in *Arabidopsis*

**FIGURE 2:**
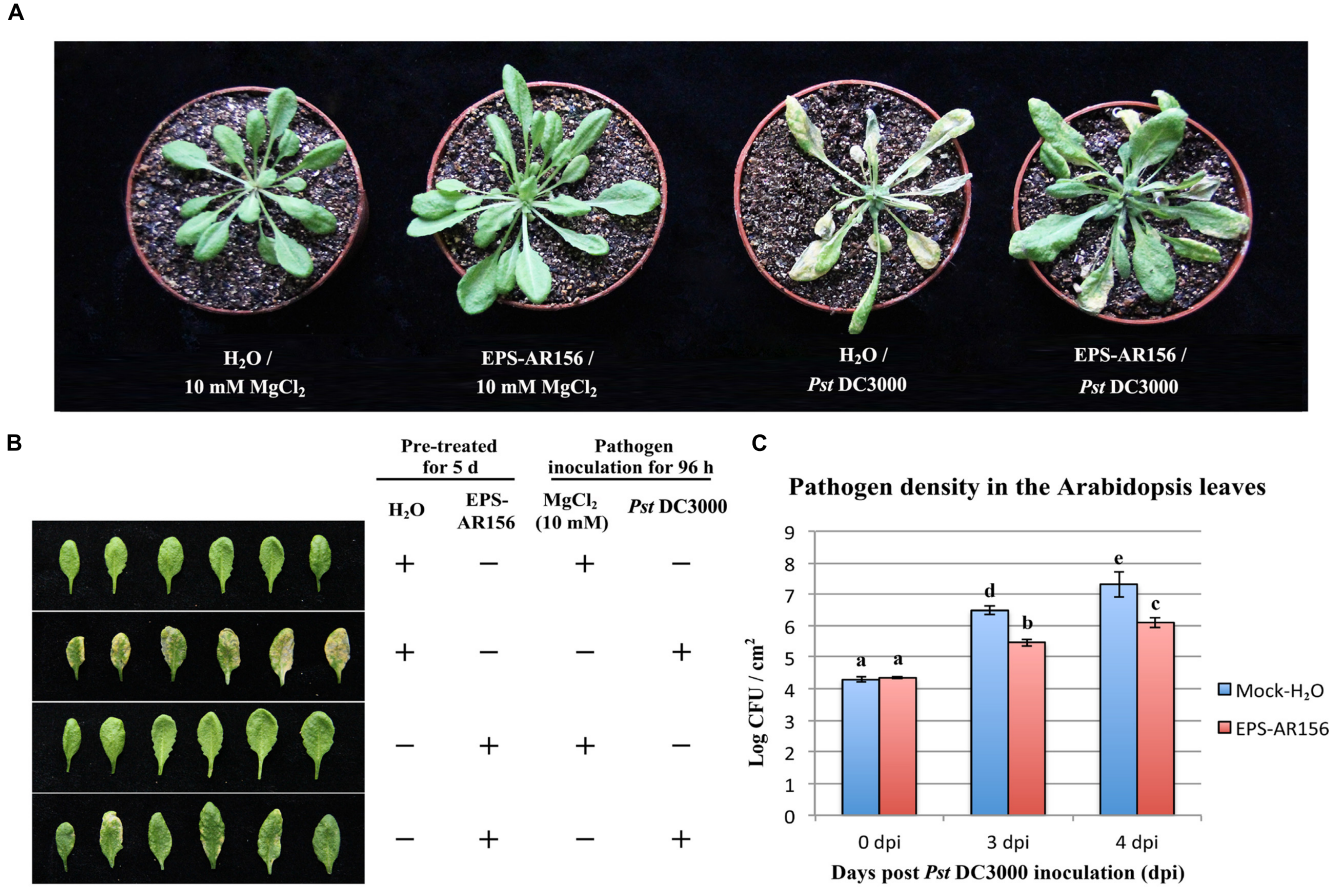
**Induction of resistance to *Pst* DC3000 by the EPSs of *B. cereus* AR156 in *Arabidopsis* ecotype Col-0 plants.** Plants were pretreated by being sprayed with *B. cereus* AR156 EPS (50 mg/ml) or sterile water (as a mock control). Five days later, leaves were sprayed with *Pst* DC3000 at 5 × 10^7^ CFU ml^-1^ or 10 mM MgCl_2_ (as a mock control). **(A,B)** Disease symptoms caused by *Pst* DC3000 infection in *Arabidopsis* plants. A representative plant from each treatment was photographed at 5 dpi. **(C)** Bacterial growth assay of *Pst* DC3000 in *Arabidopsis* treated with *B. cereus* AR156 EPS or sterile water. Data are means and standard deviations (SDs) (*n* = 24). Letters above the bars indicate statistically significant differences between treatments [least significant difference (LSD) test, *P* < 0.05]. dpt, days post-inoculation. All experiments were performed three times, and similar results were obtained.

Research has shown that flg22 can induce ROS production and enhance plant resistance to *Pst* DC3000 infection (Lu et al., 2009; Zhang et al., 2010; Lin et al., 2014). To determine whether *B. cereus* AR156 EPS 6 could act as a MAMPs and induce systemic resistance to *Pst* DC3000 infection, *B. cereus* AR156 EPS were analyzed for their ability to trigger ISR to *Pst* DC3000 in *Arabidopsis* Col-0 plants in a greenhouse trial, in which H_2_O was employed as a mock treatment. Four dpi, typical symptoms of bacterial speck disease-water-soaked spots or yellowing leaves or surrounded by extensive chlorosis was shown on the surface of the mock-treated plants leaves (**Figures 2A,B**). Moreover, compared with the controls, the pretreatment with *B. cereus* AR156 EPS caused a significant (*P* < 0.05) decrease in disease severity in *Arabidopsis* Col-0 plants (**Figure 2C**); therefore, the biocontrol efficacy of *B. cereus* AR156 in reducing the leaf speck disease caused by *Pst* DC3000 could up to 62.71% (**Supplementary Table S2**). To prove that the abovementioned result was not caused by the antagonistic effect of *B. cereus* AR156 EPS on *Pst* DC3000 in *Arabidopsis* Col-0 plants, we carried out an evaluation experiment on the antagonism of *B. cereus* AR156 and *Pst* DC3000 *in vitro*. As shown in **Supplementary Figure S2**, *B. cereus* AR156 EPS had no antagonistic effect on *Pst* DC3000. Overall, we could conclude that the *B. cereus* AR156 EPS could induce systemic resistance to *Pst* DC3000 in *Arabidopsis* Col-0 plants.

### The Extracellular Polysaccharides of *B. cereus* AR156 Induced Defense-related Gene Expression in *Arabidopsis*

**FIGURE 3:**
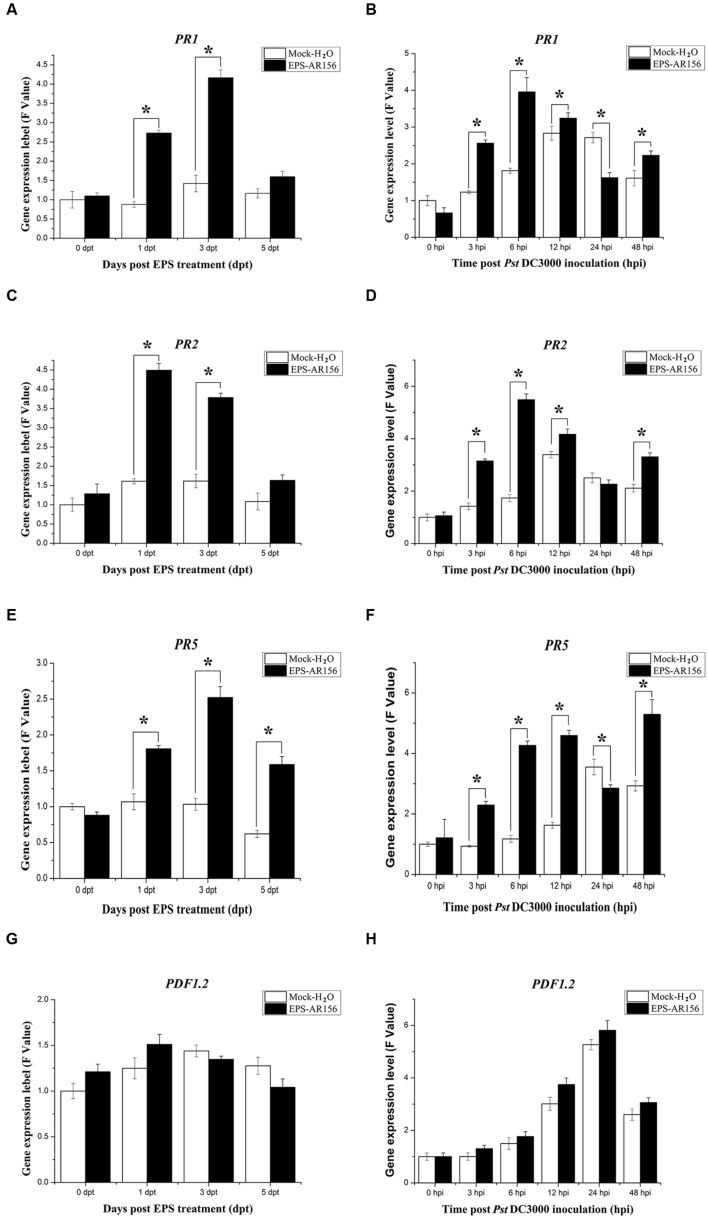
**Differential expression of SA and JA/ET signal markers in *Arabidopsis* ecotype Col-0.** Leaves of *Arabidopsis* ecotype Col-0 were harvested at the indicated time points for extracting total RNA. Gene expression levels were determined by Q-RT-PCR. **(A–H)** Time course of expression of *PR1, PR2, PR5*, and *PDF1.2* genes in the leaves of *Arabidopsis* ecotype Col-0 treated with *B. cereus* AR156 EPS alone and EPS-pretreated plants inoculated with *Pst* DC3000. The expression values of the individual genes were normalized using β-tubulin 4 as an internal standard. Data represent the average values of at least three biological replicates, each repeated in duplicate in the same run, and SDs (^∗^*P* < 0.05). dpt, days post-treatment with EPS; hpi, hours post-inoculation with *Pst* DC3000. All experiments were performed three times, and similar results were obtained.

Enhanced disease resistance in *Arabidopsis* is often accompanied by enhanced transcription of *PR* genes (such as *PR1, PR2*, and *PR5*) associated with the SA-mediated defense signaling pathway (Uknes et al., 1992; Jiang et al., 2015) and the *PDF1*.*2* gene associated with the JA/ET- mediated defense pathway. To test whether *B. cereus* AR156 EPS could induce expression levels of defense-related genes, the expression levels of all four genes were examined in plants, which were treated with *B. cereus* AR156 EPS and *Pst* DC3000 inoculation. As demonstrated in **Figure 3**, compared with the mock treatment, the transcripts of three *PR* genes were accumulated to a higher degree in the leaves treated with *B. cereus* AR156 EPS from 1 to 5 dpi and reached their maximums at 3 dpi (**Figures 3A,C,E**); however, there was no significant change in the expression level of *PDF1.2* gene (**Figure 3G**).

Q-RT-PCR was also employed to analyze the transcription of the four genes in *Arabidopsis* ecotype Col-0 plants, which were inoculated with *Pst* DC3000 alone and in those treated with *B. cereus* AR156 EPS and inoculated with *Pst* DC3000. During the pathogen challenging process, Q-RT-PCR revealed that *B. cereus* AR156 EPS stimulated the transcription of *PR1, PR2*, and *PR5* in *Arabidopsis* ecotype Col-0, which reached their maximums at 6 hpi in the samples treated with *B. cereus* AR156 EPS and inoculated with *Pst* DC3000 (**Figures 3B,D,F**). Regarding the expression levels of *PDF1.2* genes, there was no significant difference between the *B. cereus* AR156 EPS treatment and the mock treatment (**Figure 3H**). These results indicated that the transcription of the three *PR* genes (*PR1, PR2*, and *PR5*) in *Arabidopsis* treated with *B. cereus* AR156 EPS and inoculated with *Pst* DC3000 was more rapid than the treatment inoculated with *Pst* DC3000 alone. Furthermore, over all test periods, transcription of three *PR* genes (*PR1, PR2*, and *PR5*) was stronger in *Arabidopsis* treated with *B. cereus* AR156 EPS and inoculated with *Pst* DC3000 than that in *Arabidopsis* inoculated with *Pst* DC3000 alone.

### The Extracellular Polysaccharides of *B. cereus* AR156 Primes for Hydrogen Peroxide Accumulation, Callose Deposition, and Increased Defense-related Enzyme Activities in *Arabidopsis*

**FIGURE 4:**
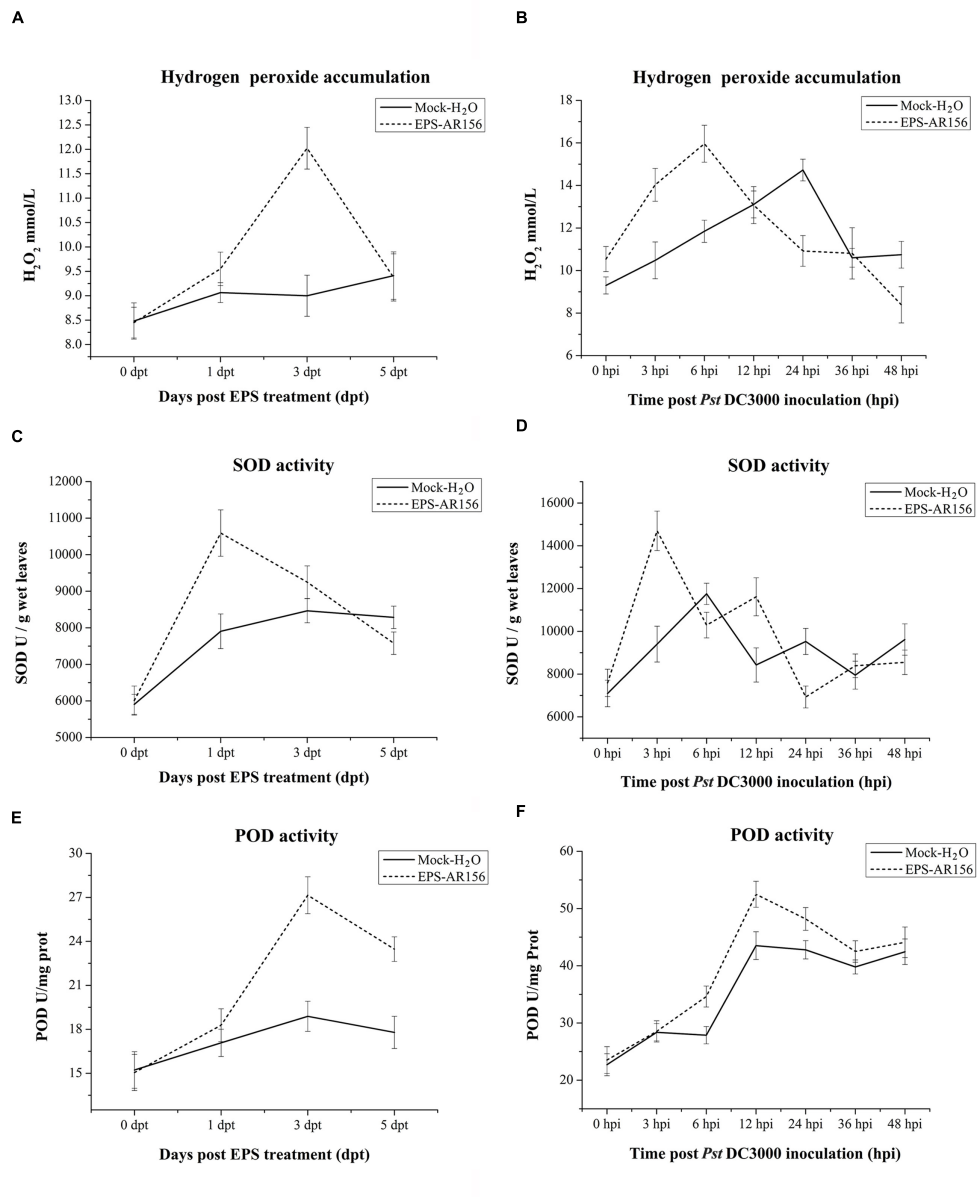
**Effect of the EPSs of *B. cereus* AR156 and pathogen challenge on hydrogen peroxide accumulation and defense-related enzyme activities in the defense responses of *Arabidopsis* ecotype Col-0.** Leaves of *Arabidopsis* ecotype Col-0 were harvested at the indicated time points for the evaluation of hydrogen peroxide accumulation and defense-related enzyme activity. **(A,B)** Time course of hydrogen peroxide accumulation; **(C,D)** SOD activity of defense-related enzymes; and **(E,F)** POD activity in the leaves of *Arabidopsis* ecotype Col-0 treated with *B. cereus* AR156 EPS for 5 days and inoculated with *Pst* DC3000. Error bars represent the standard errors of four independent treatment samples; dpt, days post-treatment; hpi, hours post-inoculation. All experiments were performed three times, and similar results were obtained.

**FIGURE 5:**
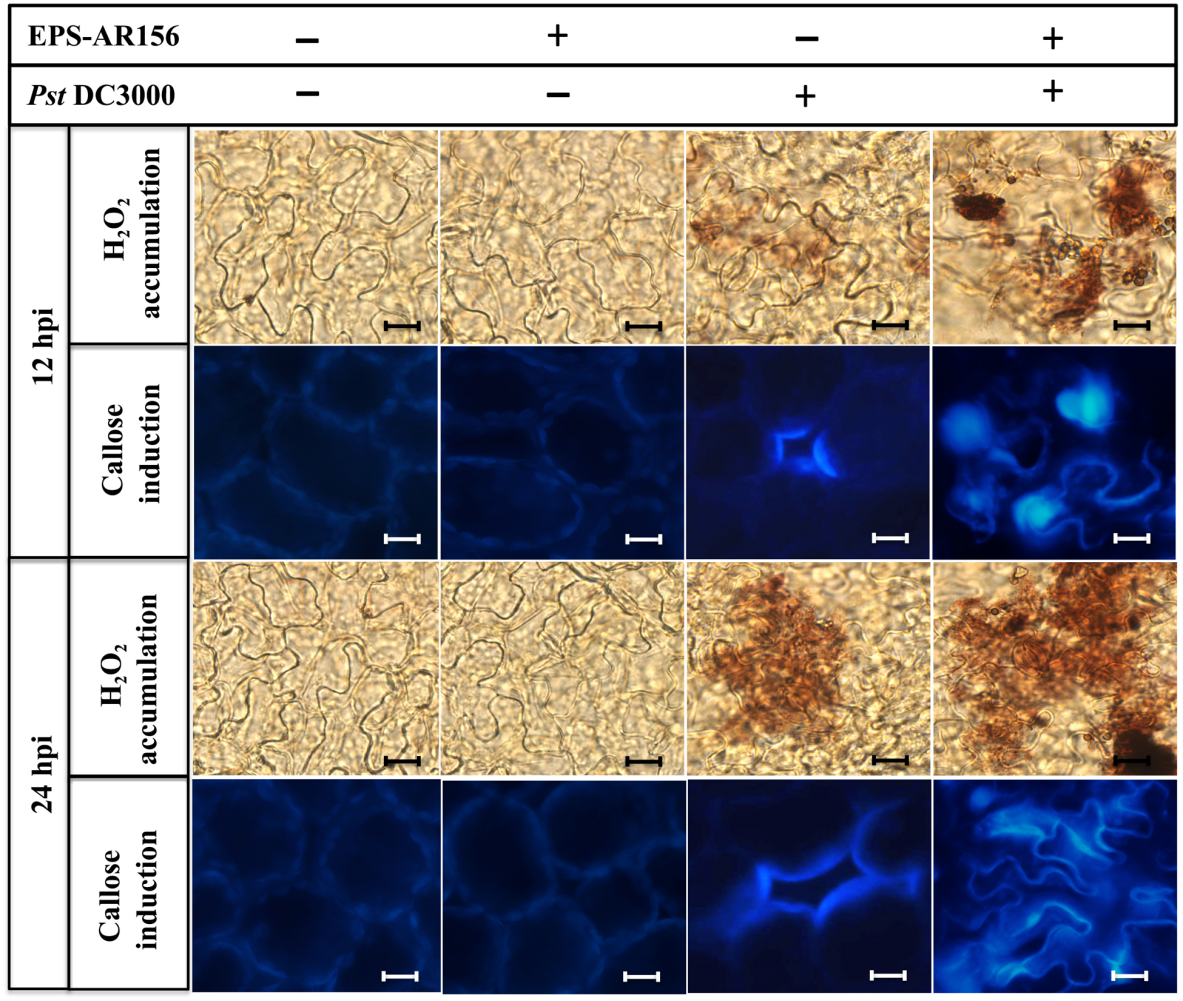
**The EPSs of *B. cereus* AR156 induced hydrogen peroxide accumulation and callose deposition in the leaves of *Arabidopsis* ecotype Col-0 plants upon *Pst* DC3000 attack.** *Arabidopsis* ecotype Col-0 plants were inoculated with *Pst* DC3000 5 days after the *B. cereus* AR156 EPS or sterile water treatment, and the leaves were sampled at 12 and 24 hpi. Hydrogen peroxide accumulation and callose deposition were observed under light and epifluorescence microscopes, respectively. Scale bars represent 20 μm. hpi, hours post-inoculation. All experiments were performed three times, and similar results were obtained.

In plants, rhizobacteria-trigered ISR is usually combined with the enhancement of activity of cellular defense responses, such as a hydrogen peroxide rapidly accumulation, callose deposition and increased defense-related enzyme activities (Conrath et al., 2002; Van Wees et al., 2008), which are response upon the pathogen attack. *B. cereus* AR156 has been demonstrated to be able to prime *Arabidopsis* for potentiated cellular defense responses (Niu et al., 2011; Jiang et al., 2015). To examine whether *B. cereus* AR156 EPS could enhance the activities of the cellular defense responses, hydrogen peroxide accumulation, callose deposition and defense-related enzyme activities in *Arabidopsis* ecotype Col-0 were detected. As shown in **Supplementary Figure S3** and **Figure 4A**, under the conditions without pathogen challenge, the accumulation of hydrogen peroxide and callose were determined at 3 dpi in the leaves of *Arabisopsis* pre-treated with *B. cereus* AR156 EPS. At 5 dpi, the change in cellular defense responses tended to decrease and stabilize, and as shown in **Figure 4A**, hydrogen peroxide accumulation peaked at 3 dpi in the leaves of *Arabidopsis* ecotype Col-0 plants treated with *B. cereus* AR156 EPS. These results are in agreement with those previously obtained from histone staining (Ref). Regarding its effect on defense-related enzyme activities, the *B. cereus* AR156 EPS treatment led to potentiated SOD and POD activities in the leaves of *Arabidopsis* ecotype Col-0 plants not challenged with *Pst* DC3000 at 3 dpt compared with the mock treatment. Under the conditions without pathogen challenge, the *B. cereus* AR156 EPS treatment increased the activities of SOD and POD at 1 and 3 dpt, respectively (**Figures 4C,E**). With pathogen challenging, he accumulation of hydrogen peroxide and callose were obviously determined at 12 hpi in the leaves of *Arabidopsis* pre-treated with *B. cereus* AR156 EPS and inoculated with *Pst* DC3000, whereas these defense responses were observed at 24 hpi in plants inoculated with *Pst* DC3000 alone (**Figure 5**). As shown in **Figure 4B**, in the leaves of *Arabidopsis* ecotype Col-0 plants, which was treated with *B. cereus* AR156 EPS and inoculated with *Pst* DC3000, hydrogen peroxide accumulation reached a maximum at 6 hpi; however, in the leaves of plants inoculated with *Pst* DC3000 alone, hydrogen accumulation reached a maximum at 24 hpi. These results are in agreement with those previously obtained by histone staining. Regarding its effect on defense-related enzyme activities, the *B. cereus* AR156 EPS treatment elevated the activities of SOD and POD at 3 and 12 hpi, respectively, (**Figures 5D,F**). These results indicate that the *B. cereus* AR156 EPS pre-treatment could make plants respond to pathogen infestation faster and more strongly by enhancing the activation of cellular defense responses.

### SA Signaling Pathways and NPR1 are Involved in the Extracellular Polysaccharides of *B. cereus* AR156 Induced Systemic Resistance in *Arabidopsis*

**FIGURE 6:**
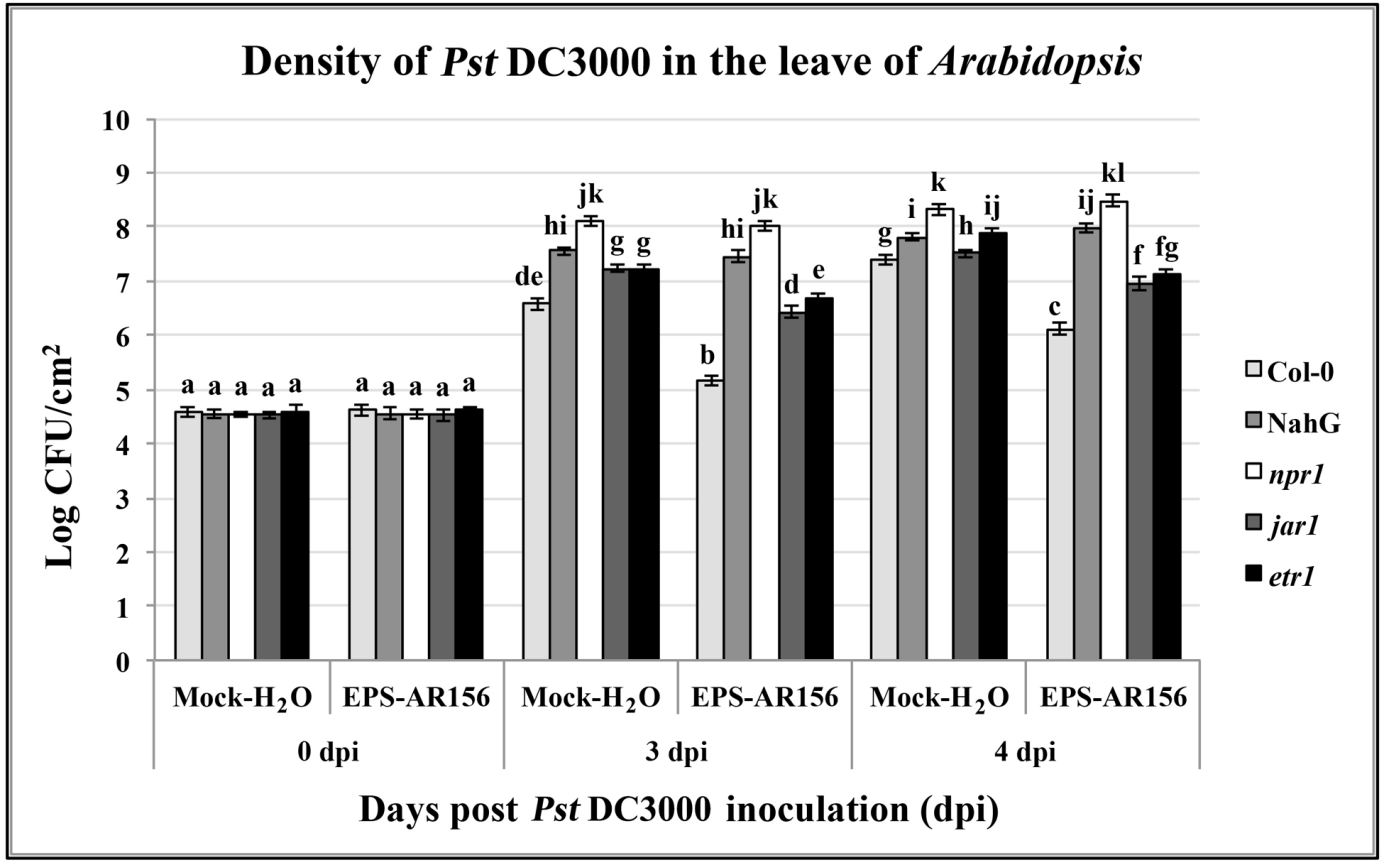
**The EPSs of *B. cereus* AR156-mediated induced systemic resistance to *Pst* DC3000 in *Arabidopsis* Col-0, NahG, *etr1*, *jar1*, and *npr1* plants at phenotypic.** Density of *Pst* DC3000 in the leaves of *Arabidopsis* plants were measured. Plants were inoculated with *Pst* DC3000 at 5 days post-treatment with *B. cereus* AR156 EPS (dpt), and leaves were harvested at 0, 3, 4, days post-inoculation. Bars represent average number of CFU per gram of leaf fresh weight. Data are means and SDs (*n* = 24). Letters above the bars indicate statistically significant differences between treatments [least significant difference (LSD) test, *P* < 0.05]. All experiments were performed three times, and similar results were obtained.

To identify the signal transduction pathways involved in *B. cereus* AR156 EPS ISR in *Arabidopsis*, an epistasis analysis method was used. We compared the levels of *B. cereus* AR156 EPS-induced resistance to *Pst* DC3000 in the wild-ecotype *Arabidopsis* Col-0, some *Arabidopsis* mutants asscociated with defense-signaling pathways, *jar1, etr1*, and *npr1* and the transgenic line NahG, and. Pre-treated with *B. cereus* AR156 EPS caused a significant (*P* < 0.05) reduction in disease severity in all tested *Arabidopsis lines* except for NahG and *npr1* compared with the respective controls inoculated with only *Pst* DC3000 (**Figure 6**).

At 3 and 4 dpi, pretreatment with *B. cereus* AR156 EPS led to a significant (*P* < 0.05) reduction in density of pathogen *Pst* DC3000 in the leaves of all tested *Arabidopsis lines* except for NahG and *npr1* compared with the respective controls inoculated with only *Pst* DC3000, indicating that the induction of ISR by *B. cereus* AR156 EPS occurred through the SA signaling pathway and was NPR1-dependent in *Arabidopsis* (**Figure 6**). These results are in agreement with those previously observed in defense-related gene expression detection components and also provide a reasonable explanation why *B. cereus* AR156 EPS could affect the transcription level of SA signaling pathway marker genes (*PR1, PR2*, and *PR5*), rather than that of the JA-responsive marker gene *PDF1.2*.

### The Extracellular Polysaccharides of *B. cereus* AR156 Trigger ISR through MAPK Signaling Pathway

**FIGURE 7:**
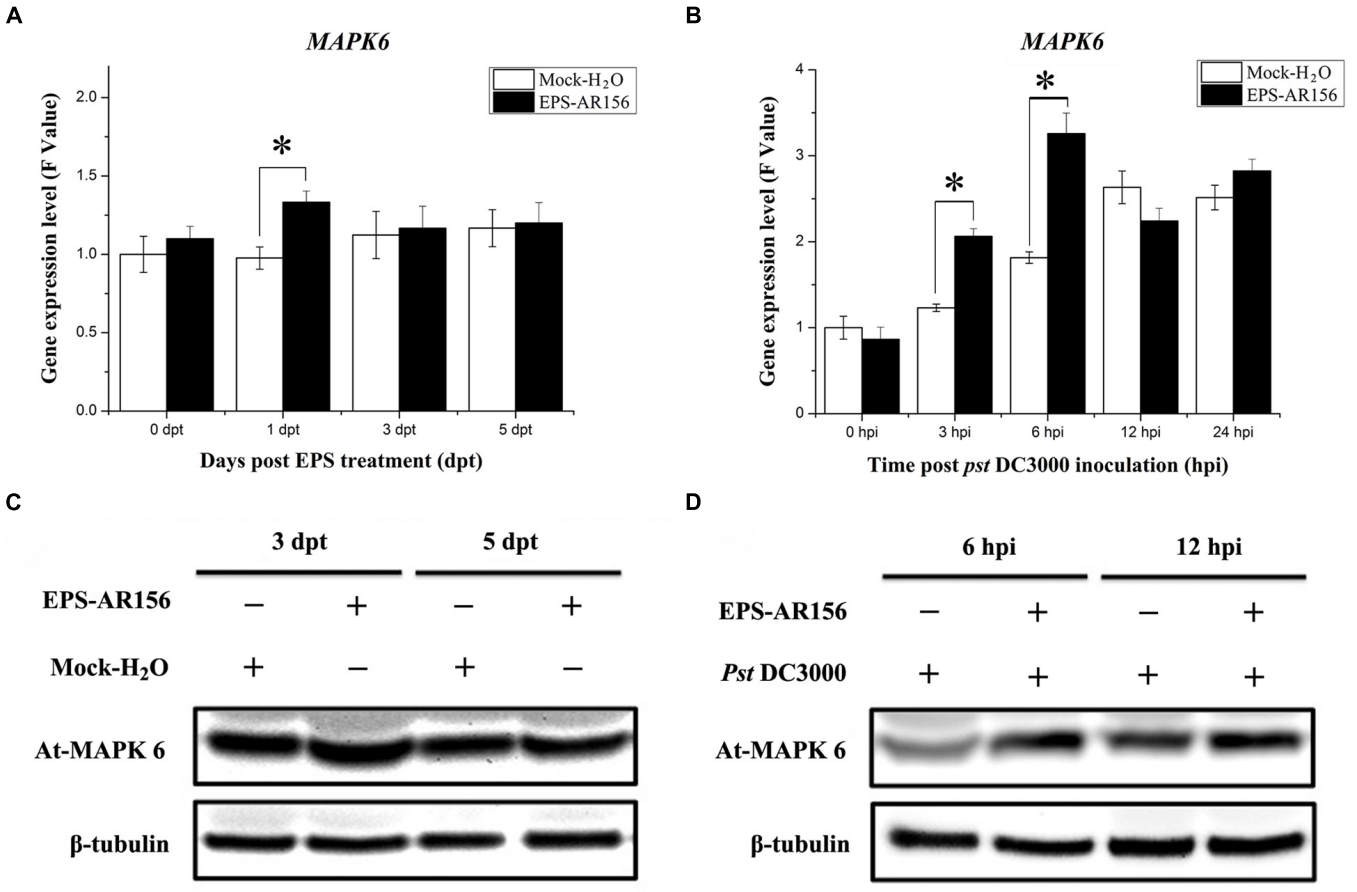
**The EPSs of *B. cereus* AR156 induced MAPK6 expression in the leaves of *Arabidopsis* ecotype Col-0 plants upon *Pst* DC3000 attack.** Leaves of *Arabidopsis* ecotype Col-0 treated with *B. cereus* AR156 EPS alone and EPS-pretreated plants inoculated with *Pst* DC3000 were harvested at the indicated time points to extract total RNA and protein; **(A,B)** *MAPK6* gene expression levels were determined by Q-RT-PCR. The expression values of the individual genes were normalized using β-tubulin 4 as an internal standard. Data represent the average values of at least three biological replicates, each repeated in duplicate in the same run, and SDs (^∗^*P* < 0.05). **(C,D)** Expression of MAPK6 in the *Arabidopsis* ecotype Col-0 treated with *B. cereus* AR156 EPS alone and EPS-pretreated plants inoculated with *Pst* DC3000 by Western blotting with a commercial MAPK6 antibody (Sigma–Aldrich), β-Tubulin was used as an internal standard. The β-tubulin antibody was purchased from Sigma–Aldrich. All experiments were performed three times, and similar results were obtained.

Microbe-associated molecular patterns or PAMPs must be perceived by PRRs (plasma-membrane localized PRRs) combined with the motivated of MAPK and trigger plant immunity (Trdá et al., 2015). For example, pathogen infection or treatments with bacterial flagellin-derived flg22 peptide, which was know as a conserved PAMP, can enhance the activation of the four *Arabidopsis* MAPKs: MPK4, MPK3, MPK6, and MPK11 (Asai et al., 2002; Beckers et al., 2009; Bethke et al., 2011). To clarify whether *B. cereus* AR156 EPS could be MAMPs and activate MAPK to trigger plant immunity, we detected the expression level of one type of *Arabidopsis* MAPK—MPK6, which is known to be activated by MAMPs—during the process of *B. cereus* AR156 EPS-ISR to *Pst* DC3000. As shown in **Figure 7**, Q-RT-PCR revealed that *MPK6* showed significantly altered expression levels in the *B. cereus* AR156 EPS-treated plants compared with the mock-treated ones and reached a maximum at 1 dpt (**Figure 7A**). During the pathogen challenging process, Q-RT-PCR revealed that *B. cereus* AR156 EPS stimulated the transcription of *MPK6* in *Arabidopsis* ecotype Col-0 and reached the maximum levels at 6 hpi in the *Arabidopsis* plants, which was treated with *B. cereus* AR156 EPS and inoculated with *Pst* DC3000 (**Figure 7B**). Moreover, the expression level of MPK6 was also detected by Western blotting with a commercial MPK6 antibody, and the results were consistent with the detection of *MPK6* in *Arabidopsis* ecotype Col-0 by Q-RT-PCR. As shown in **Figures 7C,D**, *B. cereus* AR156 EPS could enhance the expression level of MPK6 at 1 dpt, and in the leaves of *Arabidopsis* ecotype Col-0, which was treated with *B. cereus* AR156 EPS and inoculated with *Pst* DC3000, the expression level of MPK6 reached a maximum at 6 hpi (**Figure 7C**); however, in the leaves of plants inoculated with *Pst* DC3000 alone, the expression level of MPK6 reached a maximum at 12 hpi (**Figure 7C**). These results suggest that *B. cereus* AR156 EPS could be MAMPs and trigger ISR through MAPK signaling as well, and the activation of MAPK signaling in plants, pre-treated with *B. cereus* AR156 EPS and inoculated with *Pst* DC3000 show more faster and rapid than that in plants inoculated with *Pst* DC3000 alone.

### Characteristics and Composition Analysis of Extracellular Polysaccharide of *B. cereus* AR156

Investigations of the chemical compositions and molecular structures of EPS are important for establishing their function relationship. Therefore, in this study, we also analyzed the characteristics and composition of EPS extracted from *B. cereus AR156*, which could act as a MAMPs. As shown in **Supplementary Figure S4**, the UV scanning curve of *B. cereus* AR156 EPS was smooth, with a single peak at 196 nm and no absorption peak at 260 and 280 nm, suggesting that were no protein, polypeptide or nucleic acid components in the EPS samples. The IR spectral analysis results show that the EPS molecules contained different functional groups, such as hydroxyl, alkane, and carbonyl groups (**Supplementary Figure S5**). We also demonstrated that the average molecular weight of *B. cereus* AR156 EPS was approximately 13272 Da, as shown in **Supplementary Figure S6**.

After hydrolysis and anthranilic acid derivatization, the sugar composition of *B. cereus* AR156 EPS was analyzed by HPLC. The monosaccharide composition of EPS was found to consist of mannose, galactose and glucose (**Supplementary Figures S7A,B**). In terms of weight, mannose (70.97%) was found to be the major monosaccharide, followed by galactose (17.59%) and glucose (11.45%) (**Supplementary Figure S7B**).

## Discussion

In recent years, series of studies have established the functions of beneficial plant growth promotion rhizobacteria (PGPRs) on improving plant health by increasing resistance to insect pests, pathogens, and abiotic stressors, such as salinity and drought (Zhang et al., 2008, 2010; Lakshmanan et al., 2012). Among these PGPR strains, *B. cereus* AR156 could reduce disease severity when rhizoinoculated onto the roots of *Arabidopsis* plants. Niu et al. (2011) reported that AR156 inhibit the proliferation of the foliar pathogen *Pst* DC3000 through ISR, which was dependent on NPR1 and involved both the SA and JA/ET two signaling pathways. The two transcriptional factors WRKY11 and WRKY70 play roles in *B. cereus* AR156-triggered ISR to *Pst* DC3000, which explains why *B. cereus* AR156 could trigger ISR through the simultaneous activation of the SA and JA/ET two signaling pathways (Jiang et al., 2015). However, little was know on how the plant roots perceive the colonization of rhizobacteria, such as *B. cereus* AR156, and trigger ISR in response to pathogen attack. In this study, we demonstrated that the *B. cereus* AR156 EPS could act as novel MAMPs, be recognized by plants, and then trigger immunity to *Pst* DC3000 in *Arabidopsis*. We also found that perception and ISR triggering occurred through MAPK signaling and SA-signaling pathways and was NPR1-dependent.

*Arabidopsis* response to *Pst* DC3000 is ordinarily related to concomitant activation of the JA/ET defense signaling (Van Loon et al., 1998). As the results shown, the *B. cereus* AR156 EPS did not enhance the expression level of the JA/ET marker gene *PDF1.2* (**Figures 3G,H**), and pre-treatment with *B. cereus* AR156 EPS could significantly (*P* < 0.05) reduced the disease severity in *jar1* and *etr1* mutants (**Figure 6**), all these indicated that the JA/ET-dependent defense responses were not potentiated by *B. cereus* AR156 EPS upon *Pst* DC3000 infection. By contrast, *B. cereus* AR156 EPS primed the expression level of the SA-responsive genes (*PR1, PR2*, and *PR5*) upon *Pst* DC3000 invasion (**Figures 3A–F**), indicating that SA signaling might be responsible for *B. cereus* AR156 EPS-induced *Arabidopsis* resistance to *Pst* DC3000. Different bacterial PAMPs, containing flg22, could induce overlapping genes (Zipfel et al., 2004), and some studies have shown that flg22-induced gene regulation and expression are shown SA-dependent after flg22 administration (Vlot et al., 2009). In our study, we also found that the *B. cereus* AR156 EPS, by acting as novel MAMPs, could induce systemic resistance to *Pst* DC3000 by activating the SA signaling pathway, in agreement with the results of previous studies. However, in our previous study, it was found that colonization of *Arabidopsis* roots by AR156 enhanced resistance against a broad-spectrum disease and that AR156 could elicit ISR by simultaneously activating the SA and JA/ET two signaling pathways (Niu et al., 2011). However, in our study, the JA/ET-dependent defense responses were not potentiated by *B. cereus* AR156 EPS upon *Pst* DC3000 infection. Therefore, how was JA/ET-dependent signaling activated by AR156 during the ISR process? According to our results, in addition to the *B. cereus* AR156 EPS, there must be other MAMPs that facilitate perception and trigger ISR by activating the JA/ET-dependent signaling pathway.

Plants have evaluated series of mechanisms to help themselves against oomycete, fungal, bacterial, and viral infections. All these defense responses firstly start with the perception of the invading pathogen by PRRs, which can connect with PAMPs (Bittel and Robatzek, 2007; Segonzac and Zipfel, 2011). General elicitors such as flagellin (flg22), peptidoglycan (PGN), elongation factor Tu (EF-Tu), Ax21 (Activator of XA21-mediated immunity in rice), lipopolysaccharides (LPS), β-glucans from oomycetes and fungal chitin are recognized by plant membrane surface localized PRRs. Series of PAMPs and their corresponding PRRs have been identified in these years. (Newman et al., 1995, 2013; Umemoto et al., 1997; Felix et al., 1999; Lee et al., 2001; Kunze et al., 2004; Gust et al., 2007; Miya et al., 2007). Our study is the first to show that the EPSss of bacteria can act as novel MAMPs and induce visible cell death (**Figure 1** and **Supplementary Figure S1**), cellular defense responses such as ROS production and callose deposition and defense-related enzymes such as POD and SOD in the plants tested (**Figures 4** and **5** and **Supplementary Figure S3**) to trigger ISR to pathogens (**Figure 2**). These findings suggest that the *B. cereus* AR156 EPS may connect with some perception receptors localized on surface of the plant cell membrane, then trigger ISR to pathogens.

Microbe-associated molecular patterns-triggered ISR is important in assisting plants to limit pathogen growth or generate signals, which were used for adaptation to secondary infections (Coll et al., 2011; Hao et al., 2014). Many reports have documented that MAMPs, including flg22, can induce plant defense responses. However, to be able to implement this functionality, recognition by PRRs is necessary. A well-known PRR is the *Arabidopsis* receptor kinase FLS2, which can recognizes a bacterial flagellin protein (flg22), which contains a conserved 22-amino acid N-terminal sequence. FLS2 consist of an LRR domain (extracellular leucine-rich repeat), a cytoplasmic kinase domain and a trans-membrane domain (Chinchilla et al., 2006; Li et al., 2014). The LRR domain can perceive flg22 and rapidly recruits to BAK1, which was another LRR receptor-like kinase (Chinchilla et al., 2007; Heese et al., 2007; Schulze et al., 2010; Li et al., 2014). In this study, we did not identify the receptors that could recognize *B. cereus* AR156 EPS. However, to better understand the function of EPS in the interaction between plants and rhizobacteria, receptor identification will be the focus of our future studies.

However, investigations of the chemical compositions and molecular structures of EPS are important to establish their functional relationship (Jin et al., 2014). Therefore, in this study, we also analyzed the characteristics and composition of EPS extracted from *B. cereus* AR156 that could act as a MAMPs. The results of IR spectral analysis showed that the EPS molecules contained different functional groups, such as hydroxyl, alkane, and carbonyl groups (**Supplementary Figure S5**). This information will be helpful in identifying the abovementioned receptors.

Some protein kinases belong to the mitogen-activated kinases (MAPKs) family has been demonstrated as signal transduction components in a variety of processes in plants (Nühse et al., 2000). A complete *Arabidopsis* MAPK cascade consisting of MEKK1, MPK3/ MPK6, and MKK4/MKK5, may be activated in response to flg22 was identified in recent years (Asai et al., 2002). In our study, we also demonstrated that the EPS extracted from *B. cereus* AR156 could activate the MAPK cascade in response to *Pst* DC3000 infection. At the transcription and protein levels, we found that the expression of MPK6 could be increased by *B. cereus* AR156 EPS treatment in *Arabidopsis* (**Figure 7**).

**FIGURE 8:**
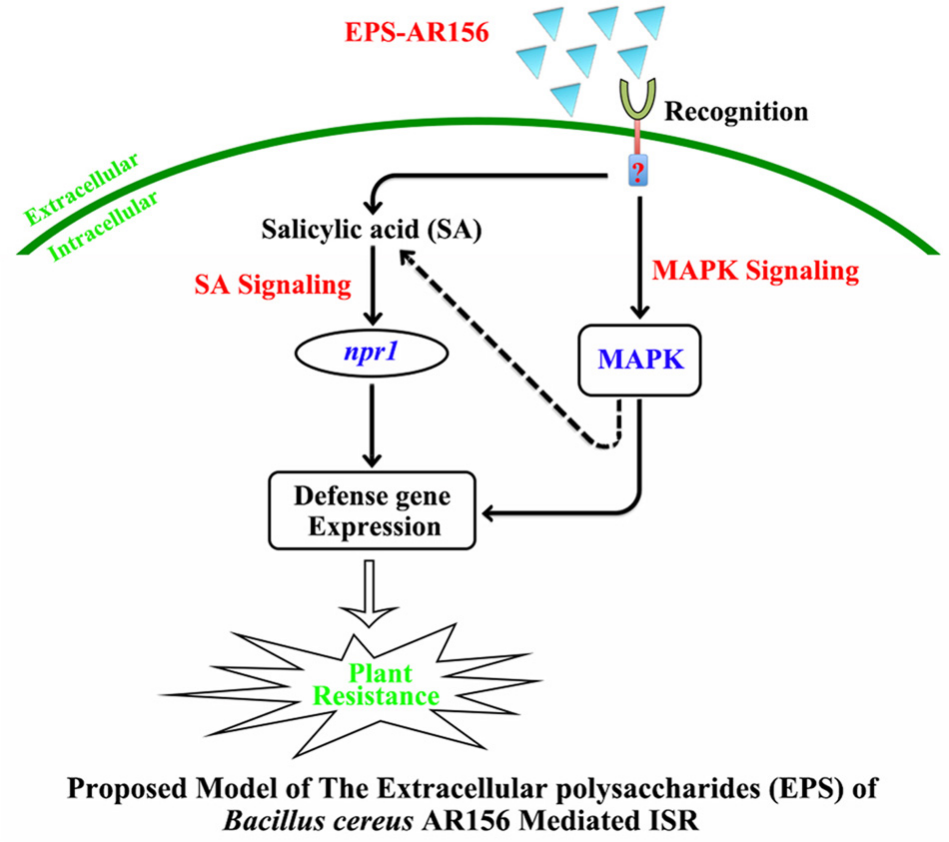
**Proposed model for the EPSs of *B. cereus* AR156 in the perception rhizobacteria AR156 by plants and triggered induced systemic resistance.** The EPSs of *B. cereus* AR156 act as unique MAMPs of AR156, and recognition by the receptors in *Arabidopsis* triggers ISR in the leaves by activating the SA-dependent signaling pathways in an NPR1-dependent manner, which leads to concurrent expression of a large set of the SA-responsive genes in the leaves and hydrogen peroxide accumulation, callose deposition and defense-related enzyme activities. Moreover, the perception of *B. cereus* AR156 EPS can also activate MAPK signaling to raise systemic resistance, which leads to the expression of MAPK6 at the transcription and post-transcription levels. This model may explain how the rhizobacteria can be recognized by plants and trigger ISR to the pathogens when localized on the surface of the plant root.

How were the *B. cereus* AR156 EPS perceived by plants, and what was their role in the process of *B. cereus* AR156-ISR? In this study, we identified *B. cereus* AR156 EPS as novel MAMPs that could be perceived by some PRRs and activated plant resistance to pathogens. A new model of the signal transduction cascade of EPS-mediated ISR in *Arabidopsis* was proposed (**Figure 8**). We found that the EPS of AR156 could be perceived by some PRRs then triggered ISR by activate the SA and MAPK signaling pathways with an NPR1-dependent manner, thus enhancing the level of induced disease resistance.

As far as we know, our study is the first to demonstrate that the EPSs of *B. cereus* AR156 can act as novel MAMPs and trigger ISR to the *Pst* DC3000 in *Arabidopsis*. Furthermore, this study is the first to illustrate how AR156 induces systemic resistance to *Pst* DC3000 in *Arabidopsis*. It is also the first to explain how the plant perceives the colonization of non-pathogenic bacteria and how rhizobacteria trigger ISR to plant pathogens when they are localized on the surface of the plant root. Future studies will focus on investigating how the EPSs are perceived by plants and how the early downstream of the plant defense response is activated and identifying the PRRs that can recognize the EPSs.

## Accession Numbers

The GenBank accession numbers for the genes mentioned in this article are as follows: *At-npr1* (NM_105102), *At-PR1* (NM_127025), *At-PR2* (NM_115586), *At-PR5* (NM_106161), *At-PDF1.2* (NM_123809), *At-MAPK6* (NM_129941), and *At-BETA-TUB4* (NM_123801).

## Author Contributions

JC-H and GJ-H designed the study. JC-H, FZ-H, and XP performed the experiments. All authors analyzed the data. JC-H and GJ-H. wrote the article. All authors contributed to the research and manuscript and read and approved the final version of the manuscript. All authors agree to be accountable for all aspects of the work.

## Conflict of Interest Statement

The authors declare that the research was conducted in the absence of any commercial or financial relationships that could be construed as a potential conflict of interest.

## Abbreviations

dpi: Day post inoculation
dpt: Day post treatment
EPS: Extracellular polysaccharides
hpi: Hour post inoculation
HR: Hypersensitive response
ISR: Induced systemic resistance
JA: Jasmine acid
MAMPs: Micro-associated molecular patterns
MAPK: Mitogen-activated protein kinases
*Pst* DC3000: *Pseudomonas syringae* pv. *tomato* DC3000
SAR: Systemic acquired resistance
SA: Salicylic acid
