# Supplementary material for: Bacillus cereus AR156 Extracellular Polysaccharides Served as a Novel Micro-associated Molecular Pattern to Induced Systemic Immunity to Pst DC3000 in Arabidopsis
Source: Front Microbiol. 2016 May 9;7:664. doi: 10.3389/fmicb.2016.00664 (PMC4876362; doi:10.3389/fmicb.2016.00664)
Supplement: Supplementary file 1 [file Data_Sheet_1.pdf]

Extracellular-polysaccharides of *Bacillus cereus* AR156 served as a novel micro-associated molecular pattern to trigger ISR to *Pst* DC3000 in *Arabidopsis*

Chun-Hao Jiang[a,1,*]; Zhi-Hang Fan [a,1,*]; Ping Xie[a,1]; Jian-hua Guo[a,1,†]

Supplementary Information

The following materials are available in the online version of this paper.

Supplementary Table 1. information of primers and gene used in this study

Q-RT-PCR Primers				
**Genes name**	**Locus**	**Forward primer**	**Reverse primer**	**Reference Paper**
At-PDF1.2	At5g44420	TCATGGCTAAGTTTGCTTCC	AATACACACGATTTAGCACC	**Journot-Catalino et al., 2006**
At-PR1	At2g14610	GGAGCTACGCAGAACAACTAAGA	CCCACGAGGATCATAGTTGCAACTGA	
At-PR2	At3g57260	CGGTACATCAACGTTGGAA	GCGTAGTCTAGATGGATGTT	
At-PR5	At1g75040	CGGTACAAGTGAAGGTGCTCGTT	GCCTCGTAGATGGTTACAATGTCA	
At-BETA-TUB 4	At5g44340	GAGGGAGCCATTGACAACATCTT	GCGAACAGTTCACAGCTATGTTCA	
AT-MAPK6	At2g43790	ATGGACGGTGGTTCAGGTCAA	TTGAAAGCAAGCGCCTCGCGG	**Menke et al., 2004**

Supplementary Table 2. Biocontrol efficacy of the extracellular polysaccharides produced by *B. cereus* AR156 to *Pst* DC3000 in *Arabidopsis*

Treatment	Diseae Severity	Biocontrol efficacy(%)
EPS-AR156	$0.335±0.0277^{b}$	62.71%
Mock-H_2O	$0.8983±0.0413^{a}$	-

Note: Values with the same English letter within the same column do not differ from each other significantly according to Duncan' test at P=0.05

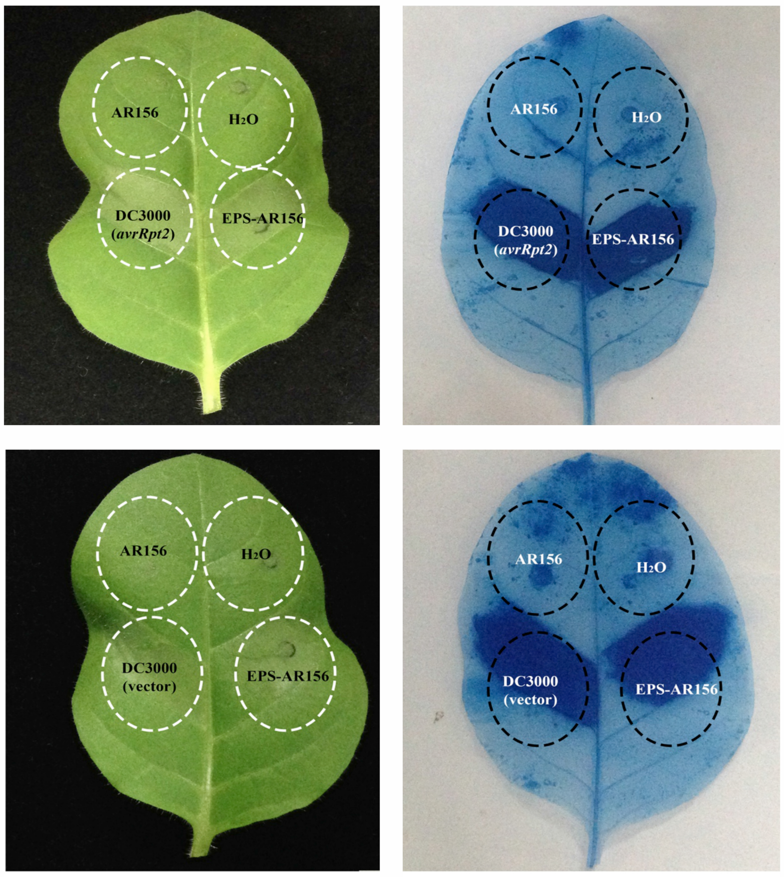

Supplementary Figure 1: The extracellular polysaccharides of *B. cereus* AR156 elicited strong HR in nonhost tobacco leaves.

The HR test by Trypan Blue staining, the leaves from 4-week-old tobacco plants were infiltrated with EPS of *B. cereus* AR156 (50 mg/ml); *Pst* DC3000 strains (1×10^7 CFU ml^{-1}) containing vector only, or clones expressing *avrRpt2*, *B. cereus* AR156 (1×10^7 CFU ml^{-1}). H_2O was employed as a negative control. Leaves were photographed by Canon EOS 700D, and then stained with Trypan Blue 3 days post-inoculation (dpi). All experiments were done three times and similar results were obtained.

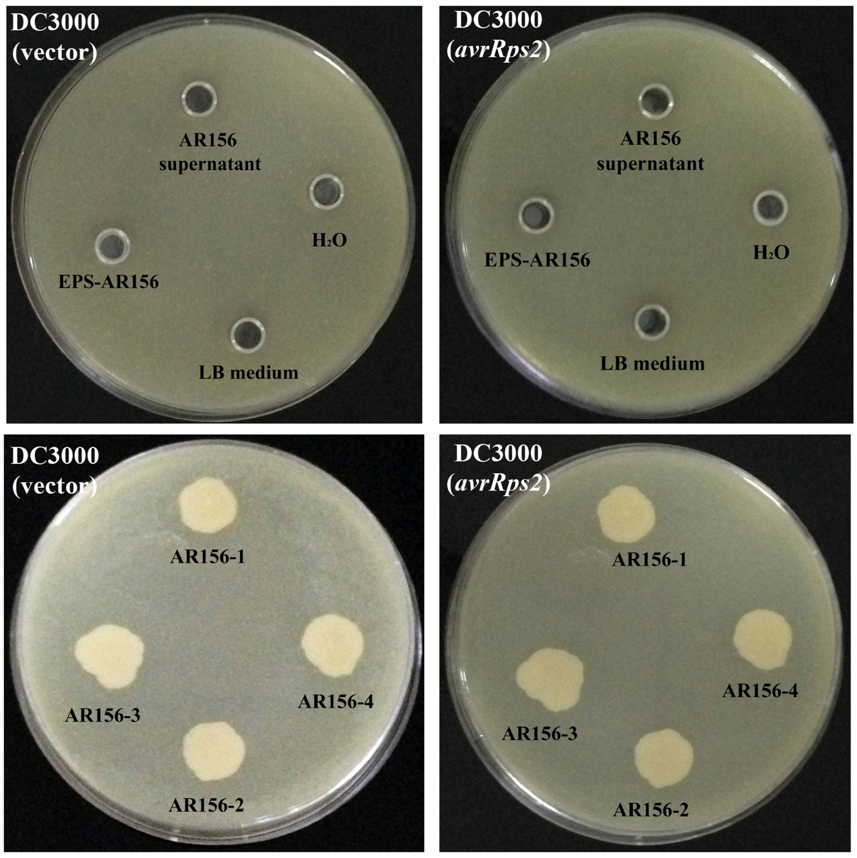

Supplementary Figure 2. The extracellular polysaccharides of *B. cereus* AR156 showed no antagonistic effect on *Pst* DC3000.

Dual culture tests for the antagonistic evaluation of the EPS of *B. cereus* AR156, *B. cereus* AR156 and its fermentation ingredients against *Pst* DC3000 strains containing vector only, or clones expressing *avrRpt2* on KB medium. Detail experimental method referenced the materials and methods.

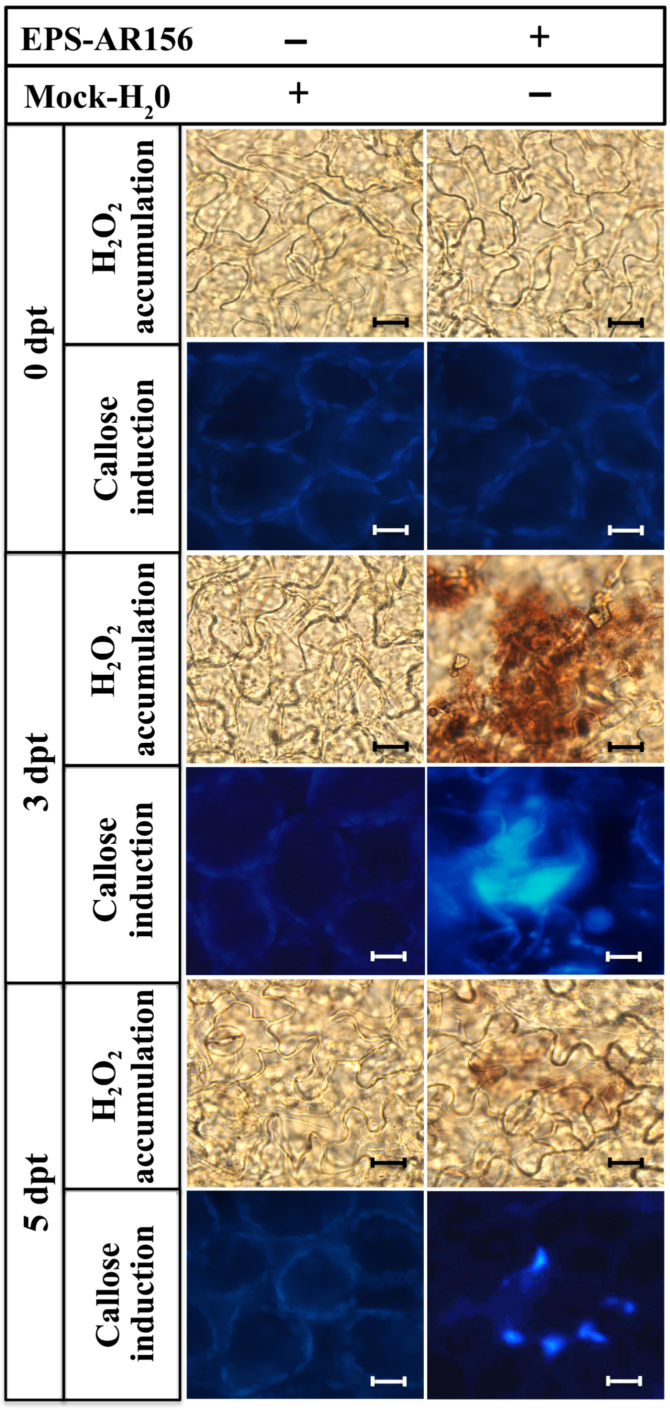

Supplementary Figure 3. The extracellular polysaccharides of *B. cereus* AR156 induced hydrogen peroxide accumulation and callose deposition in the leaves of *Arabidopsis*.

Arabidopsis ecotype Col-0 plants were treated extracellular polysaccharides of *B. cereus* AR156 or sterile water treatment, and the leaves were sampled at 0, 3, 5 dpt. Hydrogen peroxide accumulation and callose deposition were observed under light and epifluorescence microscopes, respectively. Scale bars represent 20 μm. dpt, days post-treatment. All experiments were performed three times and similar results were obtained.

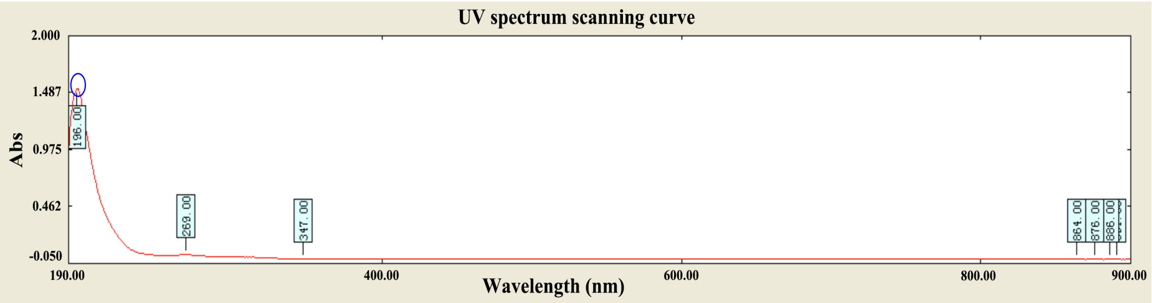

Supplementary Figure 4. The ultraviolet scanning curve of the extracellular polysaccharides of *B. cereus* AR156.

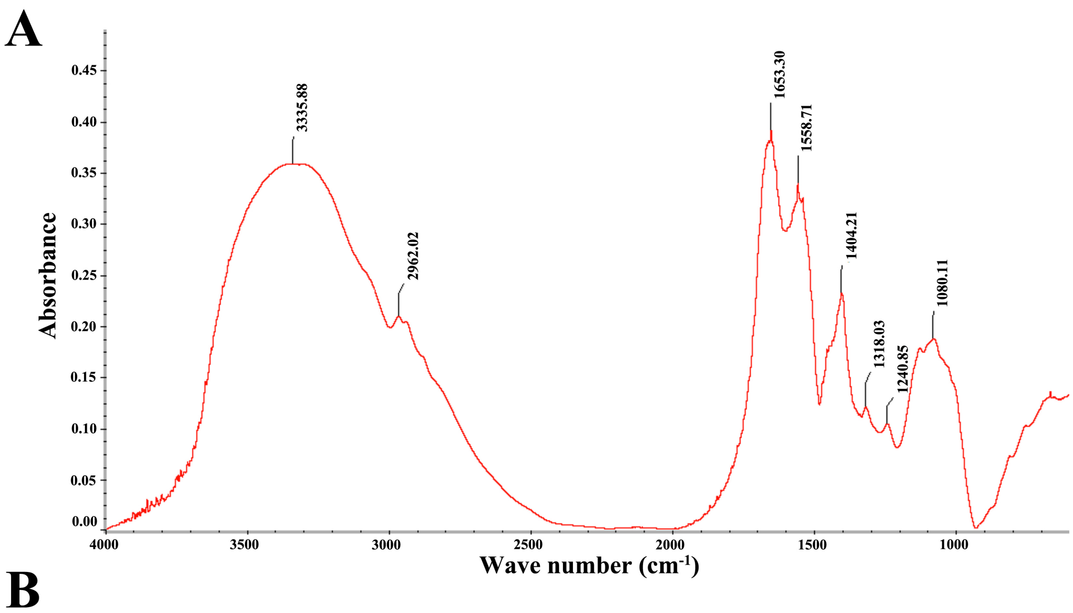

B

Absorption peak wave number（cm^{-1}）	Vibration type	Group type	Absorption peak
3335.88	ν O-H	hydroxyl	s
2962.0 nearby	ν C-H	Alkane	m
1660.29	ν -C=O	Carbonyl	s
1240.85-1558.71	δ -C-H	Alkane	m
1080.11 nearby	ν -C-C-	Alkane	m

Supplementary Figure 5. The infrared spectral analysis of the extracellular polysaccharides of *B. cereus* AR156.

(A). The infrared scanning curve of the EPS of *B. cereus* AR156; (B). Analysis of infrared spectrum scanning results. The infrared spectral analysis results show that there were serials of group types between EPS molecular, such as hydroxyl, alkane and carbonyl.

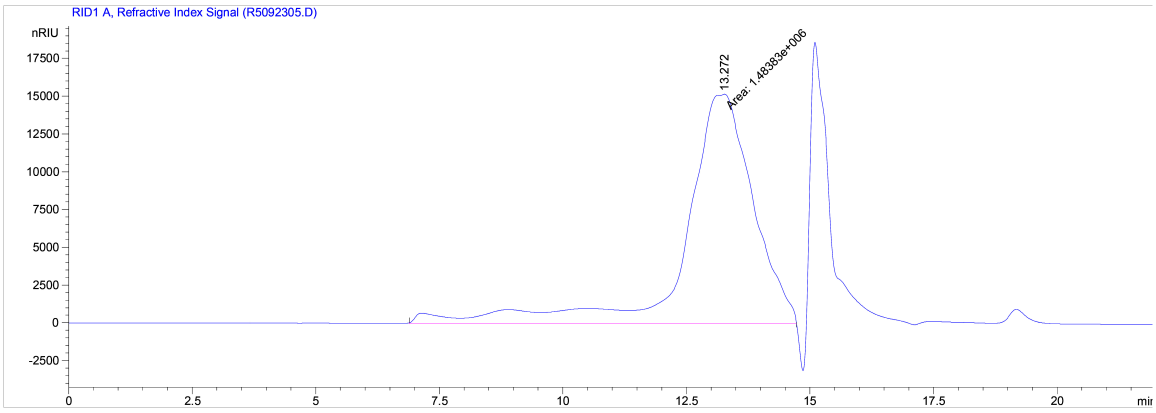

Supplementary Figure 6. Molecular weight determination of extracellular polysaccharide of *B. cereus* AR156.

A

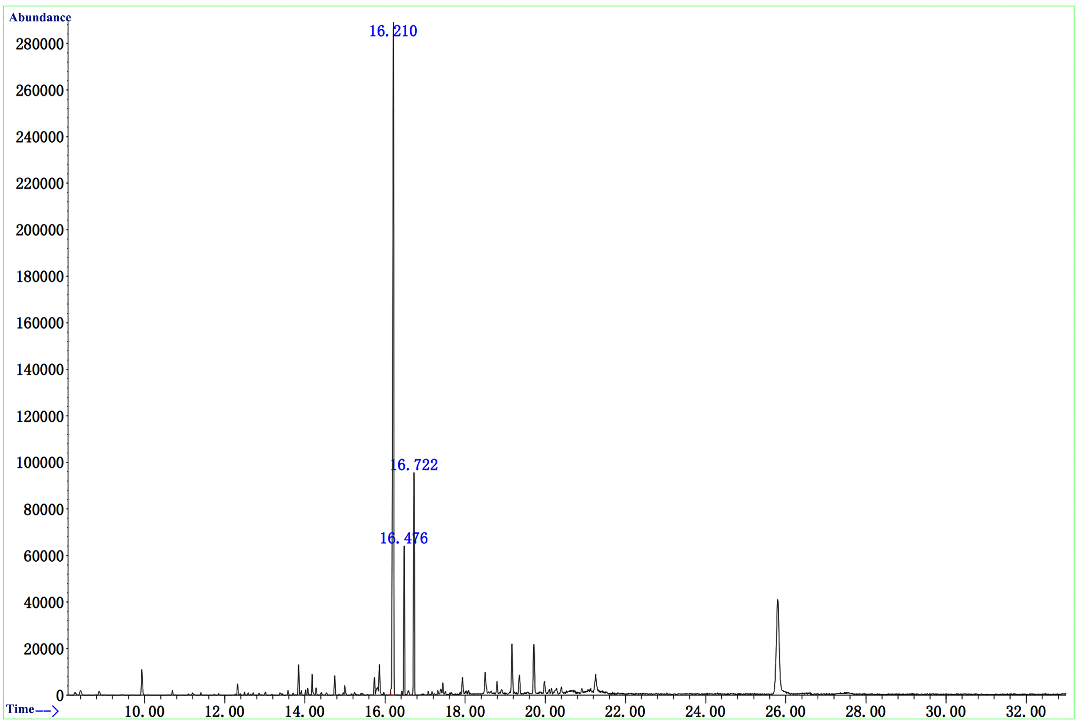

B **Analysis of the composition of the extracellular polysaccharide of *B. cereus* AR156**

Peak No.	Retention time （min）	Initial scanning	Peak scanning	Stop scanning	Peak type	Peak height	Correction Areas	Modified maximum （%）	Total content （%）	monosaccharide residues category
1	16.210	1553	1566	1572	M	298622	5711942	100.00%	70.97%	Mannose
2	16.476	1610	1617	1623	M	64541	921125	16.13%	11.45%	Glucose
3	16.722	1656	1664	1670	M	96350	1415521	24.78%	17.59%	Galactose

Supplementary Figure 7. Composition of the monosaccharides in the extracellular polysaccharides of *B. cereus* AR156

High-performance liquid chromatogram of anthranilic acid derivatives of the standard monosaccharides and monosaccharides obtained from the acid-hydrolysed EPS

of *B. cereus* AR156. (A). Chromatograms show the presence of (1) Mannose, (2) Glucose, (3) Galactose. (B). Composition of the monosaccharides and the proportion of each group.
